# Supplementary material for: Constrained inference in sparse coding reproduces contextual effects and predicts laminar neural dynamics
Source: PLoS Comput Biol. 2019 Oct 3;15(10):e1007370. doi: 10.1371/journal.pcbi.1007370 (PMC6793885; doi:10.1371/journal.pcbi.1007370)
Supplement: S1 Text — (PDF) [file pcbi.1007370.s001.pdf]

S1 Text - Constrained inference in sparse coding reproduces contextual effects and predicts laminar neural dynamics

Federica Capparelli[1*], Klaus Pawelzik[1], Udo Ernst[1]

1 Institute for Theoretical Physics, University of Bremen, Bremen, Germany

* federica@neuro.uni-bremen.de

In the following sections we first outline how to extend the generative model to encode an arbitrary number P of patches and how to formulate it in terms of continuous variables for covering the full visual field and then we briefly report the results obtained performing the contextual-modulation experiments using a different, more general configuration of the visual field.

Generalization of the model: bigger visual field

The proposed version of the model makes use of a 2-patch visual field, which is the minimal setting to investigate the co-occurrence of features in distant locations of an image. Increasing the size of the visual field is straightforward: it suffices to generalize the expression of the feature representation given by Eq. (2) and (3) (main text) as

$$\boldsymbol{b}^u = \boldsymbol{a}^u + \sum_{v=1}^{P} C^{uv} \boldsymbol{a}^v \tag{S1}$$

for $u = 1, \ldots, P$, where P represents the total number of patches that make up the visual scene. The corresponding equation for generating the image patch $\boldsymbol{s}^u$ would still read as

$$\boldsymbol{s}^u = \Phi \boldsymbol{b}^u. \tag{S2}$$

Further generalization of the model is possible, going away from discretized partition of the visual field. Denoting by Ω_a, Ω_b and Ω_s the spatial domains of the representations in $\boldsymbol{a}$ and $\boldsymbol{b}$, and the image, respectively, we could implement a continuous version of the generative model by assuming that a visual image $s(\boldsymbol{r})$, for a particular position $\boldsymbol{r} \in \Omega_S$, is obtained by linear combinations of fundamental features $\phi_i(\boldsymbol{r} - \boldsymbol{r}')$

$$s(\boldsymbol{r}) = \sum_i \int_{\Omega_b} \phi_i(\boldsymbol{r} - \boldsymbol{r}') b_i(\boldsymbol{r}') d\boldsymbol{r}' = \int_{\Omega_b} \Phi(\boldsymbol{r} - \boldsymbol{r}') \boldsymbol{b}(\boldsymbol{r}') d\boldsymbol{r}'. \tag{S3}$$

where

$$\boldsymbol{b}(\boldsymbol{r}) = \int_{\Omega_a} C(\boldsymbol{r} - \boldsymbol{r}') \boldsymbol{a}(\boldsymbol{r}')\, d\boldsymbol{r}'\,. \tag{S4}$$

To match the hypothesis made in the main text, one should finally assume the following:

a) Features ϕ_i are localized in visual space and do not extend over a maximum range $r_{\max}$

b) C is intended to capture long-range spatial dependencies extending beyond the range $r_{\max}$ of elementary features.

Contextual modulation with a bigger surround

To check how the responses of the model's units changed when using a 'bigger surround', we repeated the simulations of the three paradigms investigated in the main text using a configuration of the visual field with 4 surround-patches instead of only one. Specifically, we consider a visual field composed of five regions, a central patch plus two horizontally and two vertically aligned surround patches, as shown in Fig. S1. For this configuration, the system of differential equations (22) easily extends to

$$\begin{cases} \tau_h \dot{\boldsymbol{h}}^u = -\boldsymbol{h}^u + \Phi^\top \boldsymbol{s}^u - \Phi^\top \Phi \boldsymbol{b}^u + \boldsymbol{a}^u \\ \tau_k \dot{\boldsymbol{k}}^u = -\boldsymbol{k}^u + \boldsymbol{a}^u + \sum_{v \in \mathcal{U}(u)} C^{uv} \boldsymbol{a}^v \end{cases} \tag{S5}$$

where $\mathcal{U}(u)$ denotes the neighborhood of patch u, with $\mathcal{U}(0) = \{1, 2, 3, 4\}$ and $\mathcal{U}(u) = \{0\}$ for $u = 1, \ldots, 4$. We find that having four surround patches instead of only one does not increase the contribution of long-range connections and does not affect the agreement between our results and experimental data. The surround modulation curves we obtained are shown in Figs. S2, S3, S4.
