# Supplementary figures and images for: Constrained inference in sparse coding reproduces contextual effects and predicts laminar neural dynamics
Source: PLoS Comput Biol. 2019 Oct 3;15(10):e1007370. doi: 10.1371/journal.pcbi.1007370 (PMC6793885; doi:10.1371/journal.pcbi.1007370)

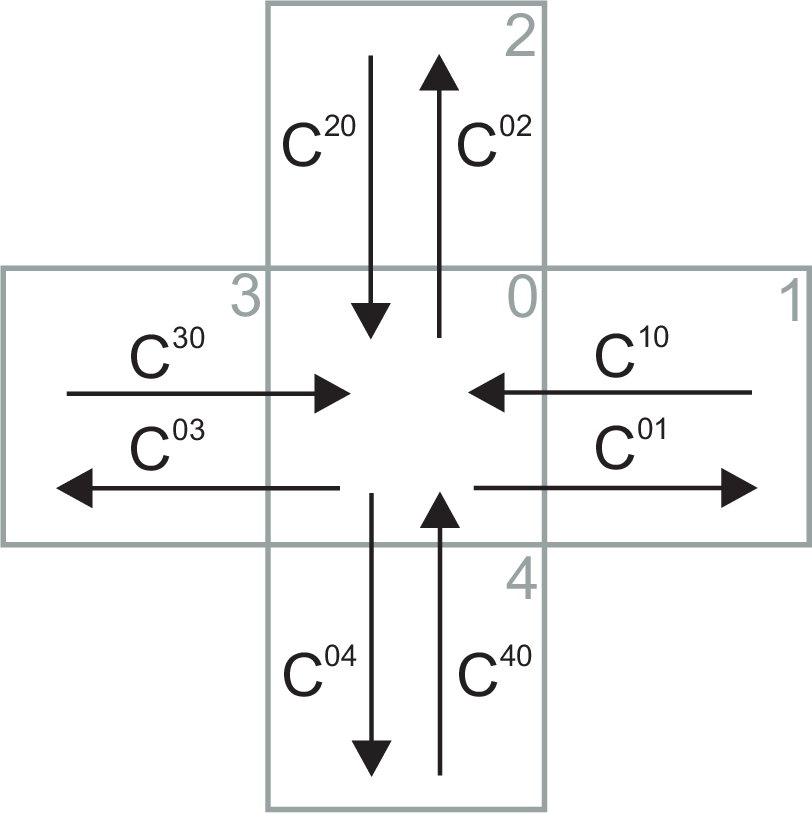

Supplement: S1 Fig — Structure of visual field used to investigate contextual phenomena, composed by one central and four surround patches. The same cross configuration is assumed for the cortical space, where Cuv denotes long-range interactions between distant regions. (TIF) [file pcbi.1007370.s002.tif]

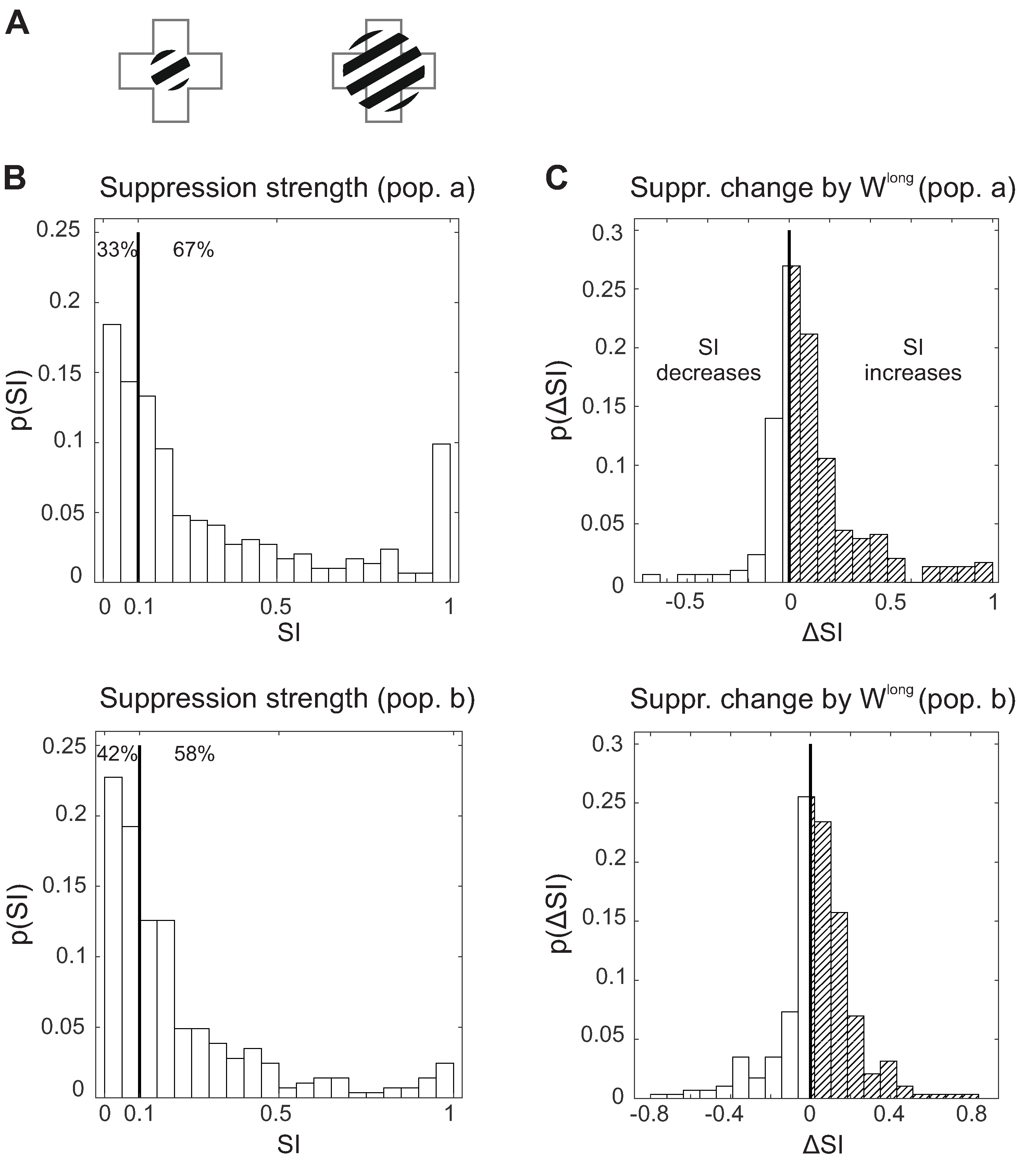

Supplement: S2 Fig — (A) Stimulus icons. (B) Distribution of suppression indices SI for the full model with long-range interactions. Values of 0 correspond to no suppression, values of 1 to full suppression. (C) Change in SI (ΔSI = SIwith long − SIwithout) induced by long-range connections. Enhanced suppression occurs more frequently than facilitation in population a and, to a lesser extent, in population b. (TIF) [file pcbi.1007370.s003.tif]

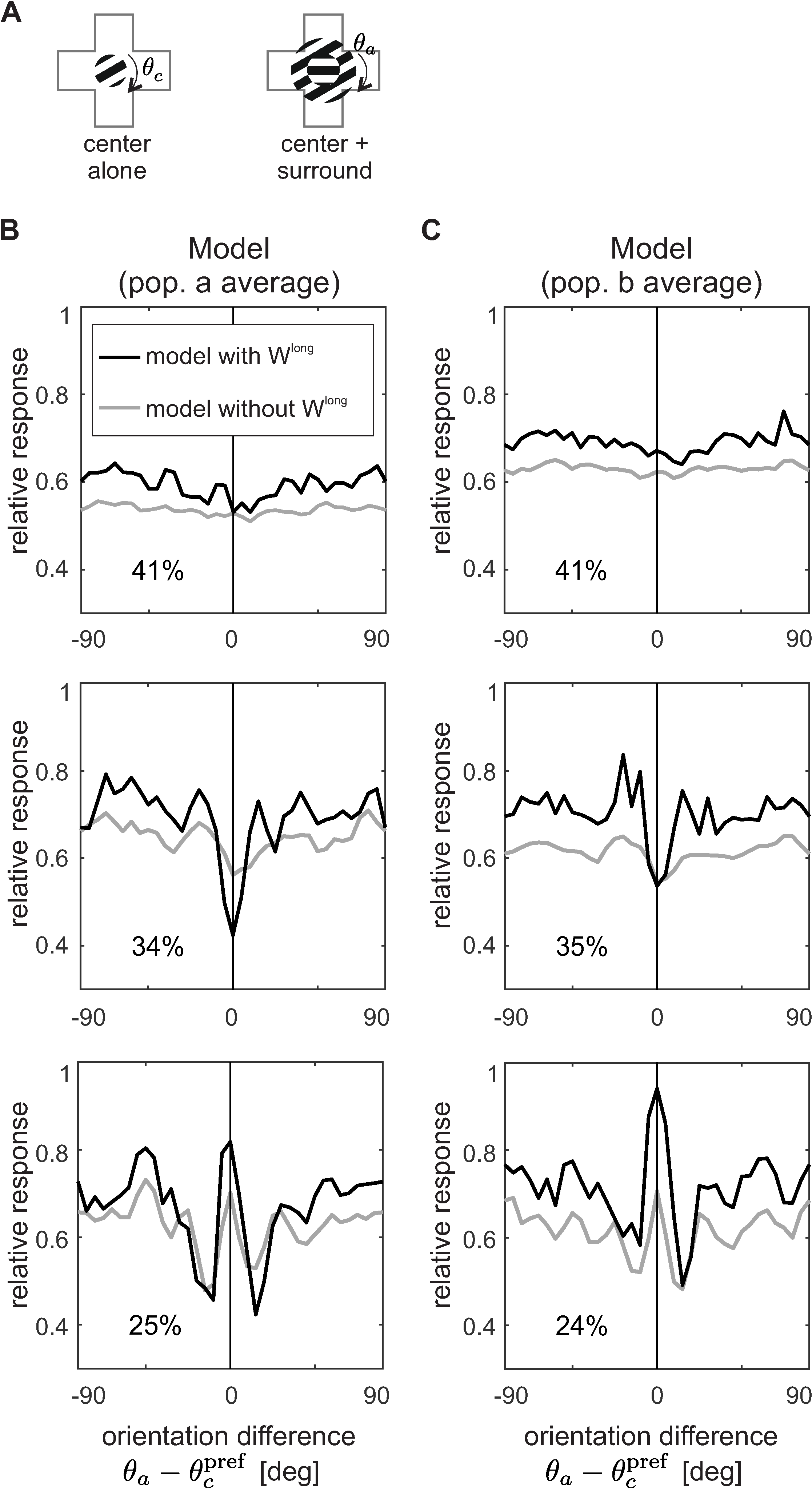

Supplement: S3 Fig — (A) Stimulus icons. (B, C) Response patterns observed experimentally reproduced by the model (from top to bottom, untuned suppression, iso-orientation suppression and iso-orientation release from suppression) in population a and b with (black curves) and without (gray curves) long-range interactions to an optimally oriented center stimulus combined with a concentric annulus of varying orientations. Note that responses are shown normalized by the response to the center alone at the preferred orientations of the units. Percentages indicate the proportion of cells that fall in the same orientation-modulation class. (TIF) [file pcbi.1007370.s004.tif]

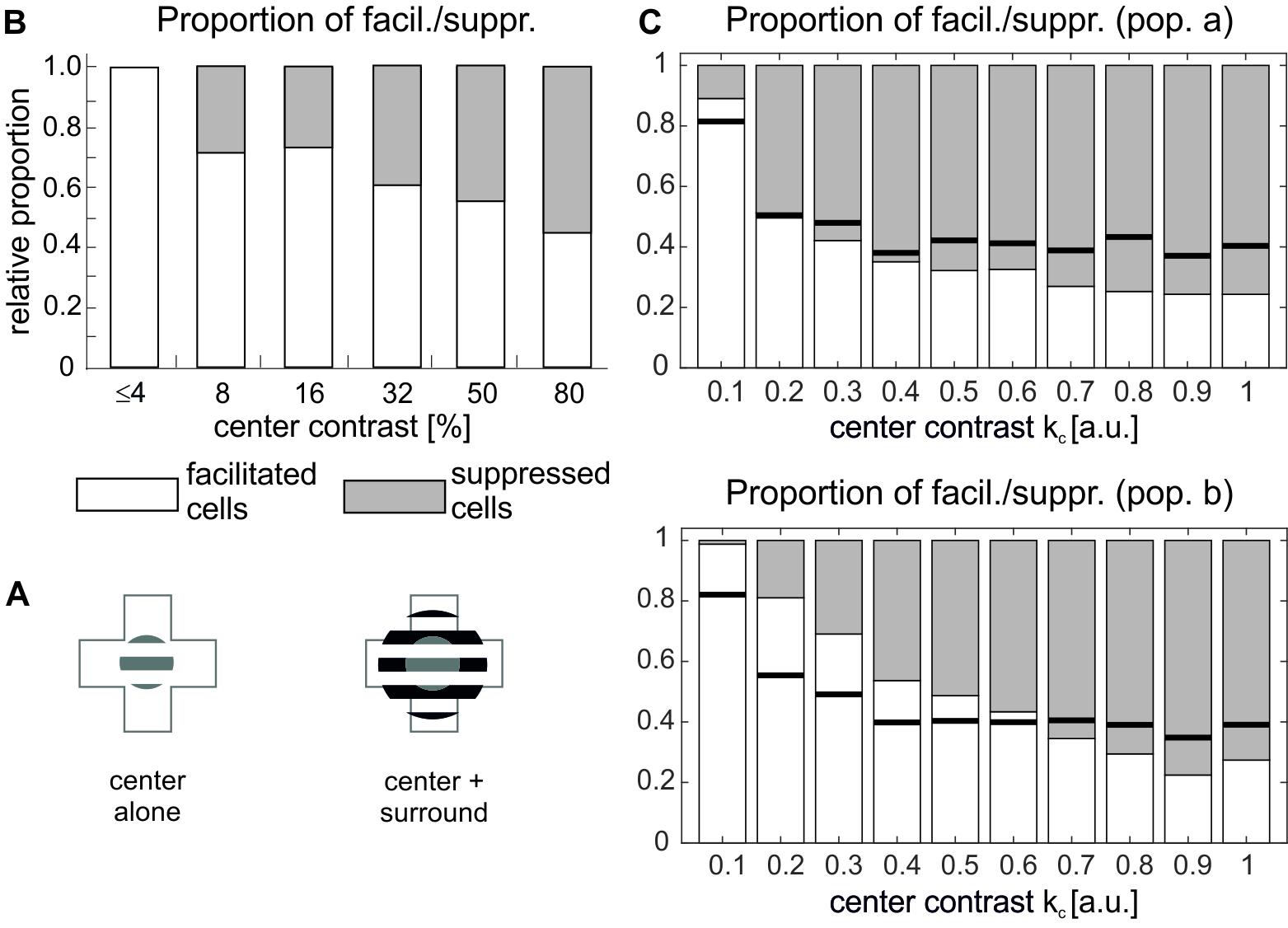

Supplement: S4 Fig — (A) Stimulus icons. (B) Population statistics, detailing the proportion of cells showing facilitation (light bars) or suppression (gray bars) in dependence on center stimulus contrast found in experiments (redrawn from [6]). (C) Population statistics computed from the model’s responses of population a (top graph) and b (bottom graph). Cells were judged to be significantly facilitated (suppressed) if their activation ratio between center-surround and center alone stimulation bsur(kc)/bcen(kc) at contrast kc was larger than 1 + ε (smaller than 1 − ε), with ε = 0.01. Solid black lines indicate proportion of cells showing facilitation without long-range interactions. (TIF) [file pcbi.1007370.s005.tif]
